# Foraging Behaviour of Juvenile Female New Zealand Sea Lions (*Phocarctos hookeri*) in Contrasting Environments

**DOI:** 10.1371/journal.pone.0062728

**Published:** 2013-05-06

**Authors:** Elaine S. Leung, Amélie A. Augé, B. Louise Chilvers, Antoni B. Moore, Bruce C. Robertson

**Affiliations:** 1 Department of Zoology, University of Otago, Dunedin, New Zealand; 2 School of Surveying, University of Otago, Dunedin, New Zealand; 3 Department of Conservation, Aquatic & Threats Unit, Wellington, New Zealand; Texas A&M University-Corpus Christi, United States of America

## Abstract

Foragers can show adaptive responses to changes within their environment through morphological and behavioural plasticity. We investigated the plasticity in body size, at sea movements and diving behaviour of juvenile female New Zealand (NZ) sea lions (*Phocarctos hookeri*) in two contrasting environments. The NZ sea lion is one of the rarest pinnipeds in the world. Most of the species is based at the subantarctic Auckland Islands (AI; considered to be marginal foraging habitat), with a recolonizing population on the Otago Peninsula, NZ mainland (considered to be more optimal habitat). We investigated how juvenile NZ sea lions adjust their foraging behaviour in contrasting environments by deploying satellite-linked platform transmitting terminals (PTTs) and time-depth recorders (TDRs) on 2–3 year-old females at AI (2007–2010) and Otago (2009–2010). Juvenile female NZ sea lions exhibited plasticity in body size and behaviour. Otago juveniles were significantly heavier than AI juveniles. Linear mixed effects models showed that study site had the most important effect on foraging behaviour, while mass and age had little influence. AI juveniles spent more time at sea, foraged over larger areas, and dove deeper and longer than Otago juveniles. It is difficult to attribute a specific cause to the observed contrasts in foraging behaviour because these differences may be driven by disparities in habitat/prey characteristics, conspecific density levels or interseasonal variation. Nevertheless, the smaller size and increased foraging effort of AI juveniles, combined with the lower productivity in this region, support the hypothesis that AI are less optimal habitat than Otago. It is more difficult for juveniles to forage in suboptimal habitats given their restricted foraging ability and lower tolerance for food limitation compared to adults. Thus, effective management measures should consider the impacts of low resource environments, along with changes that can alter food availability such as potential resource competition with fisheries.

## Introduction

Foraging behaviour can be influenced by numerous intrinsic (e.g. physiological and morphological) and extrinsic (e.g. environmental) factors [1]. Within physiological and morphological constraints, foragers exhibit plasticity under different environmental conditions and can show adaptive responses to changing conditions [2], [3]. Geographic variation in life history traits that are influenced by environmental factors (e.g. resource availability) has been observed in many taxa, including rodents [4], [5], carnivores [6], [7] and ungulates [8], [9]. Investigating this plasticity allows us to understand species-specific responses to changing environments [10]. Hence, geographic comparisons across populations of the same species in different environments are useful in defining the degree of adaptation and in characterizing the range of behavioural traits [11].

Individuals from populations that exploit lower quality habitats (e.g. food resources are less accessible, lower quality and/or scarce) may need to expend more effort foraging than conspecifics exploiting better quality habitats (e.g. Antarctic fur seals, *Arctocephalus gazella*) [12]. The effects of a poor resource foraging habitat may be amplified for juveniles that have lower tolerance for food limitation due to their smaller size [13]. Juveniles also have limited foraging ability compared to adults, due to physiological, morphological and behavioural constraints that ultimately affect their fitness and survival [14], [15]. Decreases in food availability impact on juvenile growth and ultimately result in higher juvenile mortality [16]–[18]. Furthermore, restricted foraging skills can also result in reduced juvenile survival [14], [19], [20] and has implications for population dynamics since population declines of various mammal and bird species have been partly attributed to poor juvenile survival [19], [21], [22]. Despite juveniles being an important demographic group, studies on juvenile foraging behaviour are rare in comparison to studies on adults. Consequently, investigating factors that influence juvenile foraging ability, and hence survival, is essential to understanding various impacts on the population growth of a species [23].

The New Zealand (NZ) sea lion (*Phocarctos hookeri*) provides an opportunity to examine plasticity in foraging behaviour of an understudied age class between contrasting environments, in a declining species of management concern. The NZ sea lion is one of the rarest and most highly localized pinnipeds (sea lions, seals and walruses) in the world [24]. The species is listed as “Vulnerable” by the International Union for Conservation of Nature [25] and as “Nationally Critical” by the NZ threat classification system [26]. The NZ sea lion once ranged along the entire length of the NZ coast, extending to the NZ subantarctic islands in the south [27]. This species was extirpated from the NZ mainland and hunted to near extinction in the offshore islands by the 19^th^ century [27]. At present, the majority of the species is found in the NZ subantarctic Auckland Islands (AI; 71% of total pup production; 2010 pup production 1814±39) [28]. In 1994, a small breeding population of NZ sea lions was established following a re-colonisation event on the Otago Peninsula on the NZ mainland by a single matriarch born in AI [29]. The Otago population currently only produces 4–6 pups per year [28].

Several differences have been observed between lactating females from AI vs. those from Otago. Lactating NZ sea lions at AI are one of the deepest, longest diving otariids [30]. AI females also cover greater distances during foraging trips than many other otariid species and are hypothesized to operate at or close to their physiological maximum in a marginal foraging environment [30]–[32]. In stark contrast, lactating NZ sea lions at Otago are amongst the shallowest, shortest diving otariids [33]. Otago females have small foraging ranges and short duration foraging trips compared to AI females [34] and are hypothesized to exploit more optimal habitat than AI females [34], [35]. Furthermore, adult female NZ sea lions at Otago are also larger than females at AI (125.1±7.0 kg vs. 113.8±2.9 kg, respectively) [30], [34]. AI female NZ sea lions have amongst the lowest milk fat content reported in otariids (21.3%) [36], but Otago females have milk fat content over 1.5 times higher than AI females [37].

These large disparities between the two populations of adult female NZ sea lions suggest the extreme foraging behaviour, smaller size and low milk fat content of AI females are not species-specific, but demonstrate plasticity in behaviour, morphology and physiology in response to differences in their environment. Prey species also differed between AI and Otago NZ sea lion diet [35], [38], with the main prey species at AI having lower energy content (arrow squid, *Nototodarus sloanii*, 6.3 kJ/g; octopus, *Enteroctopus zelandicus*, 3.8 kJ/g) [38], [39] than the main prey species of Otago females (barracouta, *Thyrsites atun*, 6.1 kJ/g; jack mackerel, *Trachurus sp.*, 7.6 kJ/g) [40], [41]. This suggests the food resources at AI are lower quality than at Otago [35]. These contrasts in behaviour, morphology and diet further emphasize the hypothesized low prey resources around AI [30]. The AI are likely a marginal foraging environment with limited and/or low quality prey resources given that AI rise is an iron-limited area with low levels of phytoplankton biomass and primary production [42]. Primary production in this region is predominantly limited by the combination of available light and low levels of dissolved iron [43].

Juveniles do not have as high energetic costs as lactating females [44] and their foraging behaviour is not constrained by having to return ashore periodically to nurse dependent young. Given that juveniles and lactating females face different limitations on foraging behaviour, these two groups likely adapt differently to environmental and human-induced changes in prey availability and distribution. Thus, we were interested in examining whether the significant differences in the foraging behaviour of adult female NZ sea lions at AI and Otago Peninsula were also evident in juveniles. By recording juvenile foraging behaviour in these two areas, we investigated the range of plasticity in body size, at sea movements and diving behaviour of an understudied age class in the NZ sea lion. We sought to answer: (1) Does juvenile female NZ sea lion foraging behaviour differ between two contrasting environments? and (2) Is the foraging behaviour of juvenile female NZ sea lions influenced by their mass, age and/or habitat? We hypothesized that the foraging behaviour of juvenile female NZ sea lions at AI is mainly influenced by the low resource habitat, as demonstrated by AI females expending more foraging effort (e.g. longer durations at sea, forage over larger areas, dive deeper and longer) than Otago females.

## Methods

### Ethics Statement

This study was conducted with approval from the Department of Conservation (DOC) and University of Otago Animal Ethics Committees (permit numbers DOC AEC158, DOC AEC 200, DOC AEC 174 and University of Otago AEC 28/10 and 72107001). Instrument deployments were performed under inhalant gas anaesthesia, and all efforts were made to minimize pain and suffering.

### Capture and Deployment

**Figure 1:**
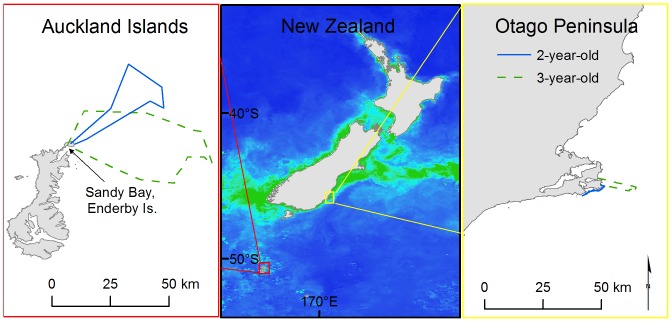
Study sites, with chlorophyll a concentration and juvenile female New Zealand sea lion satellite tracks. The scale of chlorophyll a concentration ranges from dark blue (0.1 mg/m^3^) to green (3.0 mg/m^3^). Chlorophyll concentrations calculated from ‘Ocean Colour Web’ Aqua MODIS standard mapped images (4 km resolution). The satellite tracks are from representative juvenile female New Zealand sea lions at the subantarctic Auckland Islands and Otago Peninsula, New Zealand.

We collected data over four seasons from January–February (austral summer), 2007–2010 at Sandy Bay, Enderby Island in the NZ subantarctic AI and over two seasons from March–May (austral autumn), 2009–2010 at the Otago Peninsula, South Island, NZ (Fig. 1). Logistically, we were unable to collect data at both sites during the same season because the same instruments and capture gear were used at both study sites. We addressed the potential effects of seasonal differences in the discussion.

Study animals included a sample of 2 and 3-year-old juvenile female NZ sea lions at AI and the entire known population of 2 and 3-year-old females at Otago. All animals were aged accurately based on flipper-tags that were attached within a few months after birth. We should note the logistical difficulties of deploying and recovering instruments on juveniles, a rarely seen age group (e.g. less than 4% of known-aged individuals seen over the study period at Sandy Bay were 2–3 year-old females; B.L. Chilvers, unpublished data), restricted our sample sizes at AI whereas study animals at Otago were limited by the small population size. We captured animals using a specially designed hoop net and physically restrained them with two handlers [30]. The juveniles were anaesthetised using isoflurane delivered with oxygen to a mask via a field-portable vaporiser [45] and then strapped into a custom designed restraint frame and weighed using a 200 kg capacity scale (±0.5 kg, Salter Housewares) suspended from an aluminium tripod.

Sea lions were instrumented with satellite-linked time-depth recorders (SPLASH, 100 mm×35 mm×35 mm, 150 g, Wildlife Computers, Redmond, Washington, U.S.A) or platform transmitting terminals (PTTs; Telonics 300 mW ST6, potted in epoxy, 130 mm×35 mm×15 mm, 175 g, Telonics Mesa, Arizona, U.S.A.) and time-depth recorders (TDRs; Mk9, 65 mm×18 mm×18 mm, 25 g). Sea lions were also instrumented with very high frequency (VHF; 3 cm×5 cm×2 cm, 15 g, Sirtrack, Havelock North, NZ) transmitters to facilitate recaptures. Due to equipment availability, some AI individuals were only deployed with PTTs and VHFs. We attached the instruments to the dorsal pelage of the animal below the shoulder blades on the back midline using two-part epoxy resin. Once the instruments were securely attached, we stopped the flow of anaesthetic. We measured standard body length (nose to tail) while the sea lion was allowed to recover and observed each animal until it was fully conscious. We recaptured each animal before the end of the field season to retrieve instruments.

### Satellite Data

Animal positions at sea were estimated by the Argos satellite system and assigned to different location classes based on their accuracy. However, there are large variations in accuracy of the various location classes [46]. We used different satellite data correction methods for AI and Otago data in order to obtain the best estimates of foraging ranges; our choice of method depended on the configuration of the sites and the foraging characteristics of the animals. Due to the large scale of differences found in at sea movements between AI and Otago juvenile females, using different filters did not affect the validity of the comparison between the sites. Satellite locations from animals at AI were corrected and interpolated with a state-space model, fitted into a hierarchical Bayesian context [47], [48]. We ran the models using WinBUGS [49] and R [50]. The analyses were conducted hierarchically by grouping tracks from multiple individuals within the same age group [48]. To fit the model, two Markov Chain Monte Carlo (MCMC) chains were run at a four hour time step for 40 000 iterations, with a burn-in of 20 000. To reduce sample autocorrelation, every tenth point of the remaining 20 000 samples was retained for a net of 4000 MCMC samples in each chain.

As a result of the small spatial scale of the satellite data of Otago females and based on personal observations of foraging activities of sea lions within a few meters from shore during our study, a custom-designed algorithm based on Freitas et al. [51] was used to filter Otago satellite data [34]. In addition to the Freitas et al. [51] filter (using threshold values of 3 m/s for speed and of 30° for inside turning angle), we followed these steps: (1) all locations with the highest Argos accuracy location class (i.e. LC 3) were kept during filtering, (2) locations with no neighbouring location closer than 5 km (based on a value of 4.8 km for the 95% confidence interval (CI) of nearest neighbour distances within each female’s unfiltered dataset) in the dataset of a particular animal were removed and (3) the remaining locations that were onshore but <1 km from shore were moved to the nearest location at sea [34]. To reduce sample autocorrelation, we only retained the earliest location if two or more locations were taken <30 minutes apart [34].

For AI females, we defined complete foraging trips as trips with the start and end locations within 10 km of Enderby Island [52]. At Otago, we defined complete foraging trips as trips with the start and end locations on land and the animals were at sea for more than two hours [34]. We restricted calculations of mean distance travelled per trip to complete foraging trips. Maximum distance travelled from the study site per foraging trip was measured in ArcGIS 9.3.1 [53] as the straight-line distance from the furthest recorded point to the study site, using great circle measurement type. We used filtered locations to calculate the 50% and 95% kernel utilisation distributions (UDs) using Home Range Tools [54] for ArcGIS 9.3.1. We calculated fixed kernel UDs using smoothing factors calculated from the ad hoc method (bandwidth  = 0.5) [55] and kernel UDs were used as a representation of sea lion foraging ranges [48].

### Dive Data

TDRs sampled dive depth (±0.5 m) every 5 s when wet. Depth readings were corrected for shifts in the pressure transducer at the surface of the time-depth recorders before analyses. The dive profiles were visually examined for offsets from the surface and the zero-offset correction was performed using the offset method in the package diveMove [56] in R software v2.11.1 [50]. We only analysed dives ≥3 m in depth to avoid inaccuracies in determining when sea lions were at the surface [33]. We analysed diving data by producing summary statistics for each dive using diveMove [56]. The dive summary file for each individual included the date, time, maximum depth and duration of each dive. A period ashore began after the tag was dry for 20 minutes and ended after the tag was wet for 30 seconds. Summary statistics (at sea duration and percent time spent diving, transiting and ashore) were calculated for each foraging trip.

### Statistical Analysis

We tested for mass differences between age and study site groups with t tests or Wilcoxon rank sum tests. We also derived a body condition index (BCI) from the residuals of a linear regression of mass against body length (Fig. S1) [57]. Study site differences in BCI were analysed in a linear model with study site as the predictor variable. Full morphometric data for each individual animal are provided in the Supporting Information (Table S1).

Although inter-annual variation may influence the foraging behaviour observed, small sample sizes each year precluded the differentiation of annual and individual differences. Furthermore, foraging studies on individual adult NZ sea lions followed across several years indicate little annual variability in foraging behaviour [52]. Due to limited annual sample size, we pooled the data across all years. Foraging behaviour was assessed at the scale of individual foraging trips and individual dives. Trip characteristics included at sea duration, trip distance and the maximum straight line distance travelled from the study site. Diving behaviour was characterized by the maximum dive depth and duration of each foraging trip and also by the overall mean dive depth and duration. We ran multiple linear mixed effects models using foraging behaviour characteristics as response variables and individual animal as the random effect on repeated measures data, using the R package nlme [58]. The linear mixed effects models contained five predictor variables (study site, mass, age, study site:mass and age:mass). Residual plots were examined to assess model fits and where necessary, the response variables were power, log or square root transformed to improve the normality of the model residuals.

We centred and standardized the age and mass predictor variables to improve the interpretability of regression coefficients [59]. To identify the relative importance of the predictor variables (study site, mass, age, study site:mass and age:mass) across the different foraging trip characteristics and to generate weighted coefficient estimates, we used a model averaging approach based on the Akaike Information Criterion correction for small sample size (AICc) scores [60]. We fitted a global model in R using the maximum likelihood method. A full sub-model set was generated from the global model using the R package MuMIn [61]. Models were ranked by their AICc scores and models with Δ<2 were included in the confidence model set [60]. To determine which variables had the strongest effect on foraging trip characteristics, we averaged the models using the zero method [62], where a parameter estimate (and error) of zero are substituted into models where the parameter is absent and parameter estimates are calculated by averaging over the model set [60]. We accounted for autocorrelation within individual animals (corAR1(form = ∼1|animal id)) and used a power variance function to allow for within group heteroscedasticity [63]. Lists of the model sets are provided in the Supporting Information (Tables S2 and S3).

### Primary Productivity

We assessed the primary productivity using average chlorophyll a concentration as a proxy (Fig. 1) at the two study sites during the study period (January–May, 2007–2010). We calculated mean values for each month from ‘Ocean Colour Web’ Aqua MODIS standard mapped images (4 km resolution) [64] using areas defined by the sea lions’ foraging ranges. Chlorophyll a concentrations were compared between study sites with a Wilcoxon rank sum test.

## Results

Between January–February 2008–2010, we retrieved satellite data for 19 females (seven 2-year-old and twelve 3-year-old females) and dive data for 12 females (three 2-year-old and nine 3-year-old females) at AI (Table 1). Between March–May, 2009–2010, we retrieved satellite and dive data for the five known-to-be-alive juvenile females at the Otago Peninsula (three 2-year-old and two 3-year-old females; Table 1).

**Table 1:** Sample size, trip and dive characteristics of 2 and 3 year-old juvenile female New Zealand sea lions with satellite and dive data at the subantarctic Auckland Islands and Otago Peninsula on mainland New Zealand; values are mean ± standard error of mean.

			Satellite data			Dive data			
Study site	Age(years)	Mass (kg)	Samplesize	No. of days deployed	No. of foragingtrips	Samplesize	No. of dives	Mean dive duration (min)	Maximum dive duration (min)
AucklandIslands	2	57.6±2.7	7	16.4±3.0	5.4±0.7	3	2079±526	3.1±0.7	5.2±0.3
	3	72.1±1.7	12	12.1±1.6	5.4±1.0	9	1474±262	3.3±0.5	6.5±0.3
OtagoPeninsula	2	80.5±4.0	3	35.3±2.4	31.0±2.0	3	5730±1328	1.8±0.6	6.4±1.0
	3	96.3±0.8	2	39.0±7.0	33.5±6.5	2	7828±3841	1.8±0.7	7.1±0.0

### Mass and Body Condition Index

**Figure 2:**
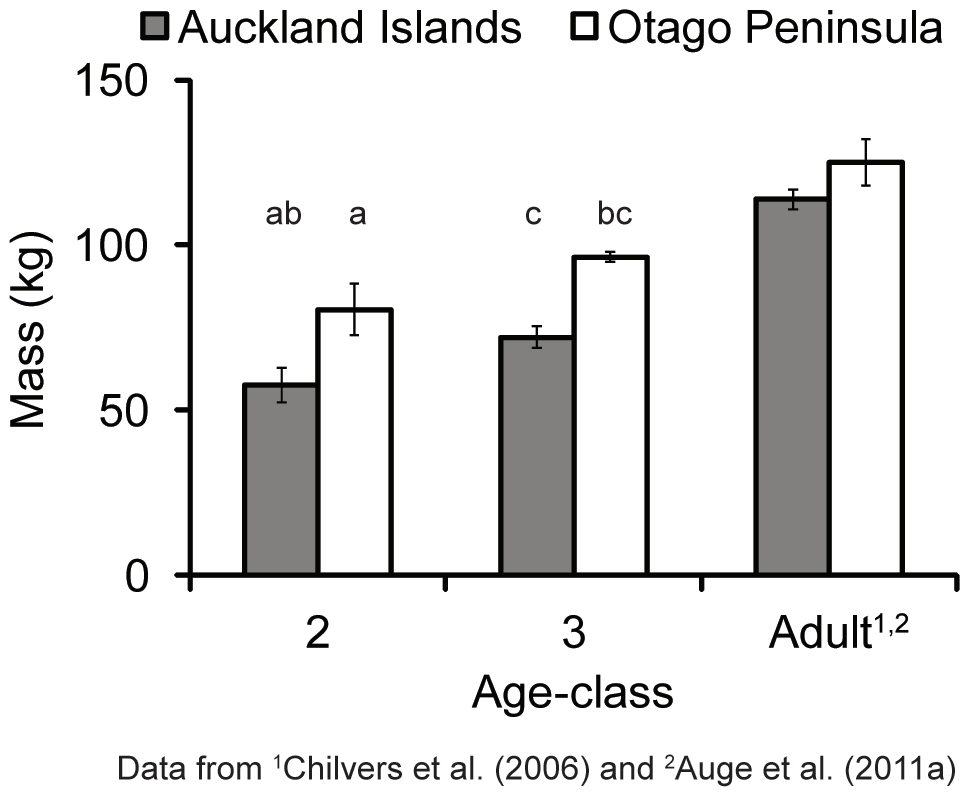
Mass of juvenile and adult female New Zealand sea lions at Auckland Islands and Otago. ^abc^Values that are significantly different from each other. Mean ± standard error of mean.

The 3-year-old juvenile females were significantly heavier than 2-year-olds at AI (t = −4.9, df  = 17, *P*<0.01; Fig. 2), but not at Otago (W  = 0, *P*  = 0.20; Fig. 2). Otago juvenile females were significantly heavier than AI animals (2-year-olds t = −4.7, df  = 8, *P*<0.01; 3-year-olds t = −5.7, df  = 12, *P*<0.01). Overall, Otago animals had better body condition than AI animals (Table S1; Fig. S1). However, there was no significant difference in BCI by study site (t  = 0.6, df  = 22, *P*  = 0.53).

### Study Site Differences in Juvenile Foraging Behaviour

Study site had the most important effect on all foraging behaviour characteristics and the effect of study site on all characteristics was large as the confidence interval for study site did not include zero (Tables 2 and 3). Both mass and age had small effects on the various foraging behaviour characteristics (Tables 2 and 3). At sea durations for AI juvenile females were three times longer than Otago females (Table 4). AI trip distances were five times longer (Fig. 1), with maximum distance travelled from the study site 10–20 times further for AI females than Otago females (Table 4).

**Table 2:** Summary results of linear mixed effects models run on juvenile New Zealand sea lion foraging trip characteristics: effects of each variable on at sea duration, trip distance and maximum distance from study site.

Trip characteristic	Variable	Estimate*	SE	Lower 95% CI	Upper 95% CI	Relative importance§
At sea duration (h; log transformed)	Auckland Islands	3.560	0.134	3.290	3.820	
	Study site	−1.100	0.280	−1.650	−0.553	**1.00**
	Age	−0.005	0.050	−0.104	0.093	0.20
	Mass	0.017	0.050	−0.138	0.172	0.23
Trip distance (km; square root transformed)	Auckland Islands	10.600	1.030	8.570	12.600	
	Study site	−6.590	1.960	−10.400	−2.740	**1.00**
	Age	−0.449	0.705	−1.830	0.932	0.48
	Mass	0.335	0.844	−1.320	1.990	0.37
Max distance from study site (km; power transformed)	Auckland Islands	1.620	0.033	1.560	1.680	
	Study site	−0.471	0.068	−0.604	−0.337	**1.00**
	Age	−0.003	0.011	−0.025	0.019	0.18
	Mass	−0.011	0.026	−0.061	0.039	0.47
	Study site:mass	−0.021	0.049	−0.117	0.075	0.23

* Effect sizes have been standardised following Schielzeth (2010). Estimate values for ‘Study site’ indicate the difference in trip characteristics between study sites.

§ Relative importance values in bold indicate the confidence intervals for these parameter estimates do not include zero, indicating these predictor variables have a strong effect on foraging behaviour.

SE, standard error; CI, confidence interval.

**Table 3:** Summary results of linear mixed effects models run on juvenile New Zealand sea lion dive characteristics: effects of each variable on dive depth and duration.

Dive characteristic	Variable	Estimate*	SE	Lower 95% CI	Upper 95% CI	Relative importance§
Dive depth (m; logtransformed)	Auckland Islands	4.220	0.055	4.110	4.330	
	Study site	−1.530	0.095	−1.710	−1.340	**1.00**
	Age	−0.001	0.017	−0.034	0.032	0.17
	Mass	−0.019	0.044	−0.106	0.068	0.38
	Study site:mass	0.023	0.071	−0.116	0.162	0.17
Maximum dive depth (m; log transformed)	Auckland Islands	5.060	0.104	4.850	5.260	
	Study site	−1.620	0.144	−1.900	−1.340	**1.00**
	Age	0.015	0.038	−0.059	0.089	0.27
	Mass	0.028	0.057	−0.083	0.139	0.34
Dive duration (min)	Auckland Islands	3.267	0.106	3.050	3.467	
	Study site	−1.448	0.195	−1.833	−1.068	**1.00**
	Age	0.028	0.059	−0.087	0.143	0.31
	Mass	0.039	0.080	−0.118	0.195	0.32
Maximum dive duration (min)	Auckland Islands	6.033	0.605	4.850	7.217	
	Study site	−1.817	0.913	−3.600	−0.022	**1.00**
	Age	0.012	0.330	−0.633	0.658	0.43
	Mass	0.500	0.433	−0.350	1.350	0.84
	Study site:mass	0.193	0.498	−0.783	1.172	0.30
	Age:mass	−0.058	0.172	−0.393	0.277	0.13

* Effect sizes have been standardised following Schielzeth (2010). Estimate values for ‘Study site’ indicate the difference in trip characteristics between study sites.

§ Relative importance values in bold indicate the confidence intervals for these parameter estimates do not include zero, indicating these predictor variables have a strong effect on foraging behaviour.

SE, standard error; CI, confidence interval.

**Table 4:** Trip and dive characteristics of 2 and 3-year-old juvenile female New Zealand sea lions at the Auckland Islands and Otago Peninsula; values are means ± standard error of mean (SEM).

	2-year-old females		3-year-old females		Overall mean	
Trip or dive characteristic	Auckland Islands	Otago	Auckland Islands	Otago	Auckland Islands	Otago
At sea duration (h)	36.4±6.7	11.6±2.9	33.7±5.0	12.0±3.6	34.7±4.0	11.8±2.3
Trip distance (km)	119.1±19.1	19.8±10.1	93.7±13.3	16.6±10.7	103.0±10.9	18.5±7.7
Max. distance from study site per foraging trip (km)	37.1±6.3	2.8±0.8	32.3±4.5	1.6±0.6	34.1±3.7	2.3±0.5
Dive depth (m)	73.4±6.2	14.2±1.2	67.8±3.4	15.8±1.6	69.2±2.9	14.8±0.9
Max. dive depth per foraging trip (m)	141.6±23.7	30.6±2.3	214.1±16.5	33.6±3.1	153.9±13.8	31.8±1.9
Dive duration (min)	3.1±0.2	1.8±0.2	3.3±0.1	1.9±0.2	3.2±0.1	1.8±0.1
Max. dive duration per foraging trip (min)	4.7±0.5	4.3±0.5	5.8±0.3	4.9±0.6	5.5±0.3	4.5±0.4

**Figure 3:**
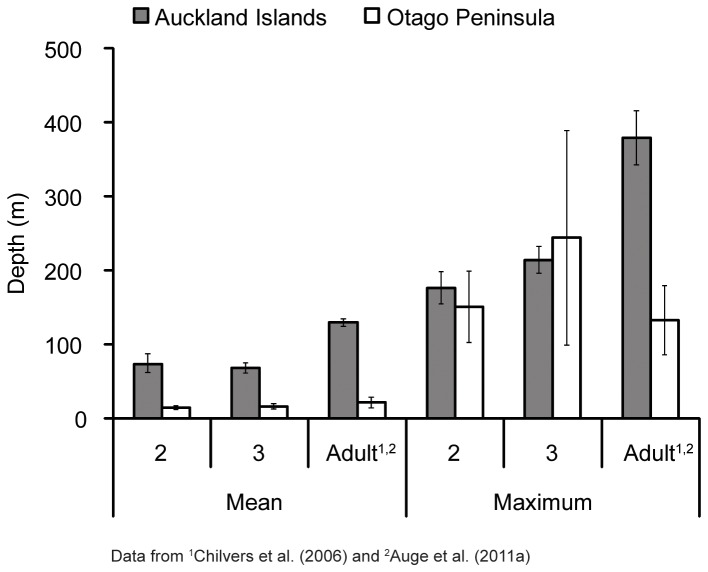
Mean and maximum dive depths for juvenile female New Zealand sea lions at Auckland Islands and Otago. Mean ± standard error of mean.

**Figure 4:**
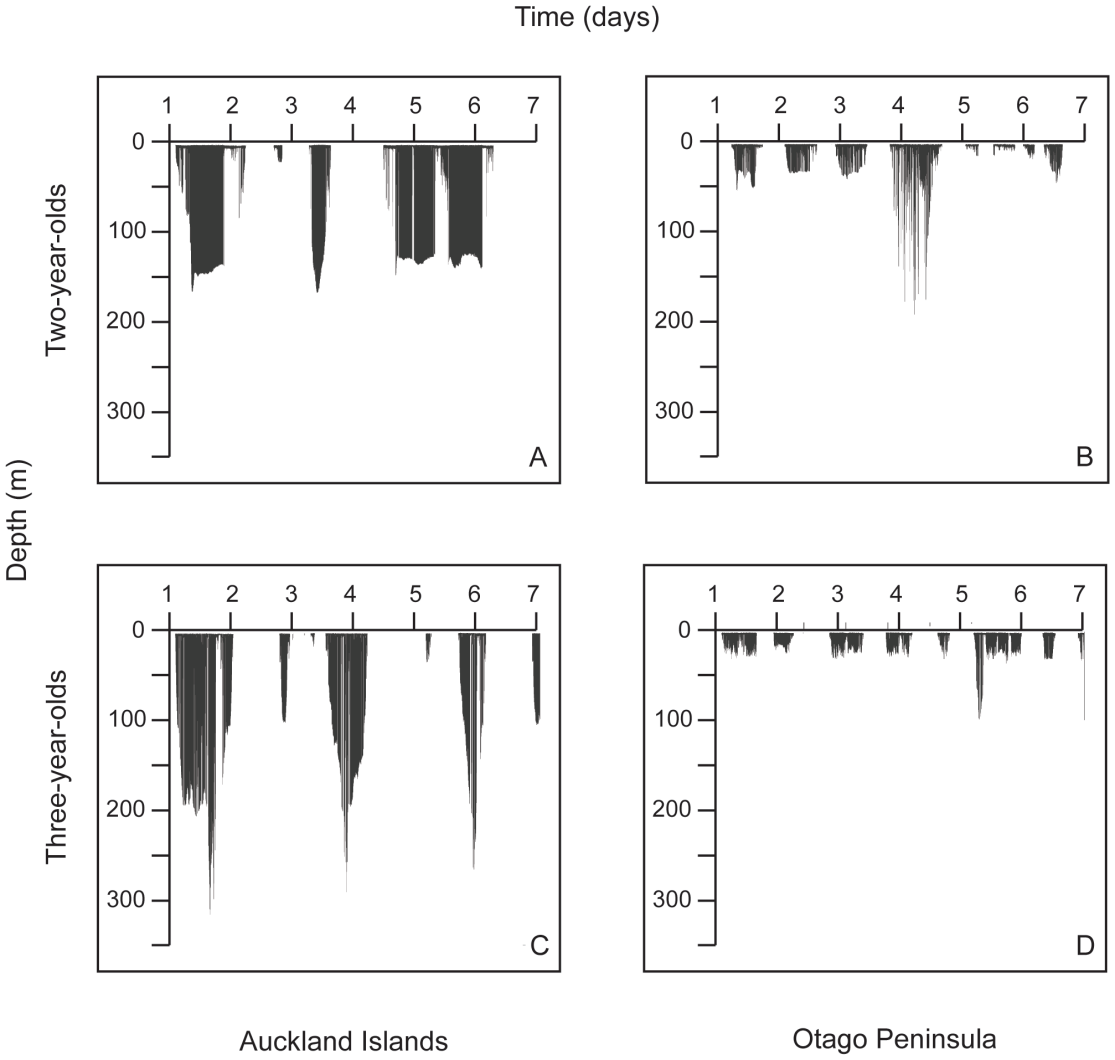
Dive profiles of representative juvenile female New Zealand sea lions at Auckland Islands and Otago. Juvenile females: A) 2-years-old Auckland Islands, B) 2-years-old Otago, C) 3-years-old Auckland Islands and D) 3-years-old Otago.

AI juvenile females dove longer and deeper than Otago females (Tables 3 and 4; Figs. 3 and 4). Average dive durations for AI females were ∼1.5 times longer than Otago durations although the maximum dive durations reached were within a similar range for both sites (Table 4). Average dive depths were approximately four times deeper for AI females than Otago females; however, the maximum dive depths reached were within a comparable range for both sites (Table 4; Fig. 3). However, dives >100 m represented only 1% of Otago juvenile dives compared with 33% of AI juvenile dives.

### Primary Productivity

The average chlorophyll a concentration was significantly higher at the Otago Peninsula (1.73±0.84 mg/m^3^) than the AI (0.43±0.12 mg/m^3^) for the duration of the study period (W  = 319, *P*<0.01). The yearly averages ranged from 0.41–0.45 mg/m^3^ at AI and 1.21–2.48 mg/m^3^ at Otago from 2007–2010.

## Discussion

Juvenile NZ sea lions demonstrated plasticity in both body size and foraging behaviour in contrasting environments. AI juvenile female NZ sea lions were smaller (Fig. 2) and expended more foraging effort (i.e. spent more time at sea, foraged over larger areas and also dove deeper and longer; Figs. 1 and 3) than Otago juveniles. Juvenile female NZ sea lions were lighter than adult females, with AI juveniles ∼51–63% of the mass of adults and Otago juveniles ∼65–76% of the mass of adults (Fig. 2). AI juveniles reached maximum depths and durations that were only 50% of adult female levels (Fig. 3) and did not access adult female foraging grounds (Chilvers et al. 2005, 2006, E. Leung, unpublished data), likely limiting their available foraging habitat [30], [32]. In contrast, Otago juvenile female NZ sea lions dove to the same depths (Fig. 3) and durations as Otago adult females [33] and exploited adult female foraging grounds [34], due to the relatively shallow dive depths and short trip distances of Otago adults compared to AI adults. Differences in foraging behaviour were mainly due to study site, rather than mass or age influences (Tables 2 and 3). However, there are several mechanisms that may contribute to the observed contrasts in foraging behaviour, such as differences in habitat/prey characteristics, conspecific density levels or interseasonal variation. Our results mirror the observed differences in body size and foraging behaviour of adult NZ sea lions at AI and Otago and corroborate the hypothesis that AI are less optimal habitat than Otago [30], [32]–[34].

### Habitat Effect on Juvenile Foraging Behaviour and Body Condition

Although foraging behaviour is constrained by numerous factors, the distribution of a predator’s foraging is largely influenced by the distribution of their prey. The short trip distances and durations of Otago females suggest there is sufficient food near-shore for them. However, predators will need to increase foraging effort, search time and search area (e.g. increase dive frequency, foraging trip duration and distances) when there is lower prey abundance around the central place [12], [65]–[67]. The larger foraging area, longer trip durations and deeper dive depths at AI imply prey resources are scarcer and less accessible, as was suggested with Antarctic fur seals at Heard Island [12]. The hypothesis that AI has lower prey resources is supported by the lower levels of primary productivity in AI sea lion foraging areas compared to Otago. Furthermore, the difference in diet (i.e. prey species) between NZ sea lion populations [35], [38] suggest that prey availability differs between sites. Increased travel costs (e.g. further distance to foraging grounds, deeper depths or greater vertical movement) need to be offset by higher energy gains [68]. Despite higher travel costs, AI animals exploit less energy dense prey than Otago individuals [35], [38], [39]. This indicates that AI individuals are expending more foraging effort for lower energetic payoffs (e.g. less profitable prey) than Otago animals. The increased foraging effort may be a reflection of the behavioural plasticity (e.g. foraging strategies) required for successful foraging of different prey types and may indicate an adaptive trait in a species that is experiencing fluctuating conditions. It is important to note that although diet composition differed between AI and Otago populations, diet was similar between juvenile and adult NZ sea lions at each site [35], [37], [38].

The mass of AI study animals were within the range of mean mass calculated for 2 and 3-year-old female NZ sea lions in a larger study [69] and thus indicates that we did not inadvertently sample only smaller individuals. Nevertheless, Otago female NZ sea lions were heavier than AI females, for both juveniles and adults (Fig. 2) and had better body condition (Fig. S1). However, these animals exhibited diving behaviour contrary to what was expected based on their size. Mass and diving ability are positively correlated in diving mammals and birds, with larger animals diving deeper and longer than smaller individuals [70]. Interestingly, the smaller AI juvenile females on average exhibited extreme diving behaviour compared to the larger Otago juveniles (Table 4; Figs. 3 and 4), despite Otago individuals having access to deep water (depths >200 m) within 20 km of Otago. Furthermore, Otago juvenile females demonstrated the ability to dive to similar maximum depths and durations as AI individuals (Fig. 3, Table 4), suggesting that there are no differences in physiological capacity between the two populations in their ability to exploit similar depths. The larger mass of Otago animals is likely a reflection of the increased food resources in this region, especially given that the original recolonizing female at Otago was from AI and thus the Otago population is not from a different genetic stock than AI.

### Effects of Differences in Competition Levels and Season

Although differences in habitat-related effects such as prey availability may influence the observed contrasts in juvenile NZ sea lion foraging behaviour, confounding factors such as differences in conspecific density levels or season may also impact foraging behaviour. For instance, AI individuals may face higher intraspecific competition because ∼10,000 NZ sea lions inhabit the NZ subantarctic islands [71], compared to ∼170 NZ sea lions at Otago (McConkey, pers. comm.). Thus, AI individuals may need to expend greater foraging effort in response to higher competition for prey resources. However, while the NZ sea lion populations at AI have declined over the last 15 years, the foraging behaviour of adult females there has not changed over this period [30]. This trend suggests that NZ sea lion foraging behaviour at AI is likely not constrained by intraspecific competition [34]. Furthermore, intra and interspecific competition also exist at Otago since there are numerous marine predators that forage around Otago, including ∼20 000 NZ fur seals (*Arctocephalus fosteri*) [72], which feed on the same prey species and in similar areas as NZ sea lions [73]. Thus, differences in competition levels likely have less influence than habitat-related effects on the large contrasts in foraging behaviour between females at Otago and AI.

Seasonal differences in prey distribution may also explain differences in foraging behaviour between AI and Otago individuals since data were collected in the austral summer and autumn, respectively. For example, prey may be distributed at deeper depths or farther locations during the summer and at shallower depths and closer to colonies during the autumn. Hence, it is possible that juvenile NZ sea lions may exhibit more similar foraging behaviour in the same season. However, diet studies of both populations in the autumn have found AI and Otago animals forage on different prey types in the same season [35], [38], with AI animals mainly targeting prey (e.g. arrow squid) found from surface waters to 500 m depth [74], while Otago individuals largely exploit prey (e.g. barracouta and jack mackerel) found from near the surface to 200 m depth within the continental shelf [75]. Thus, disparities in foraging behaviour are likely due to differences in prey behaviour at the two sites, with the main prey of NZ sea lions at AI being distributed at deeper depths than at Otago. Furthermore, primary productivity is higher at Otago than AI across all seasons [64], suggesting higher resources at Otago year-round. Hence, similarly, it is likely that the observed differences in foraging behaviour are more strongly driven by habitat-related effects than seasonal differences.

### Implications of Suboptimal Habitat for Conservation Management

Low food availability can have large impacts on population dynamics since unfavourable environmental conditions can have immediate effects on animal performance (e.g. lower survival or fecundity) [76]. Our study provides further support for Augé et al’s [34] conclusion that current population management models probably overestimate the rate of population increase of NZ sea lions by modeling populations exploiting optimal habitat. Moreover, fisheries operations around AI may further reduce prey resources in this foraging environment [77]. The predominant human threat to the NZ sea lion at AI is the commercial arrow squid trawl fishery, both through mortality from bycatch and potential resource competition [28]. Juveniles are more vulnerable than adults to decreases in prey availability due to their restricted foraging ability [76] and thus, are likely more susceptible to resource competition. Resource competition with fisheries has been reported for numerous pinniped species [78]–[80] and is also likely for the NZ sea lion [77]. However, management measures to mitigate NZ sea lion-fishery interactions only focus on reducing bycatch rates in the squid fishery and do not consider the potential impacts of resource competition [81]. Given the continued decline of the nationally critical NZ sea lion, effective management measures should also consider the impacts of potential resource competition and the suboptimal habitat of AI.

### Conclusions

Juvenile NZ sea lion foraging behaviour differed between study sites, with age and mass having little influence on foraging behaviour. Otago juvenile and adult female sea lions are larger and feed on higher energy prey [35] with less foraging effort [30], [33] in a higher productivity area, than AI juveniles and adult females. The plasticity in morphology and foraging behaviour exhibited in juvenile and adult NZ sea lions may be an adaptive trait in a species that is experiencing fluctuating conditions (e.g. differences in food availability and prey behaviour). However, we cannot attribute the contrasts in NZ sea lion foraging behaviour to a specific cause because various mechanisms (e.g. differences in competition levels or season) may also influence the observed differences in foraging behaviour. Nevertheless, the deeper dive depths and farther trip distances of AI juvenile NZ sea lions suggest prey are less accessible and scarcer at AI than at Otago. The combination of greater foraging effort, smaller body size, lower energy diet and lower productivity at AI suggest AI are less optimal habitat (e.g. lower quality and less prey resources) than Otago. It is likely more difficult for juveniles to successfully acquire food in suboptimal habitat given their restricted foraging ability compared to adults. This work is critical information for the management of NZ sea lions and our results support the suboptimal habitat hypothesis. When this is added to the known low juvenile survival and low reproductive rates of AI NZ sea lions [82], [83], this emphasizes that management of this species needs to be precautionary.

## Supporting Information

Figure S1 **Relationship between mass and body length of juvenile (2 and 3-years-old) female New Zealand sea lions (** ***Phocarctos hookeri*** **) and a definition of the body condition index (BCI) as the residual value between observed and expected mass.**
(DOC)

Table S1 **Morphometric, dive and satellite data available for individual juvenile female New Zealand sea lions (** ***Phocarctos hookeri*** **) at Auckland Islands (AI) and Otago Peninsula. BCI, body condition index.**
(DOC)

Table S2 **Results of linear mixed effects models run on juvenile New Zealand sea lion (** ***Phocarctos hookeri*** **) foraging trip characteristics: at sea duration, trip distance and maximum distance from study site.**
(DOC)

Table S3 **Results of linear mixed effects models run on juvenile New Zealand sea lion (** ***Phocarctos hookeri*** **) dive characteristics: mean and maximum dive depth and mean and maximum dive duration.**
(DOC)
