# Supplementary material for: Foraging Behaviour of Juvenile Female New Zealand Sea Lions (Phocarctos hookeri) in Contrasting Environments
Source: PLoS One. 2013 May 6;8(5):e62728. doi: 10.1371/journal.pone.0062728 (PMC3646001; doi:10.1371/journal.pone.0062728)
Supplement: Figure S1 — Relationship between mass and body length of juvenile (2 and 3-years-old) female New Zealand sea lions ( Phocarctos hookeri ) and a definition of the body condition index (BCI) as the residual value between observed and expected mass. (DOC) [file pone.0062728.s001.doc]

Figure S1. Relationship between mass and body length of juvenile (2 and 3-years-old) female New Zealand sea lions and a definition of the body condition index (BCI) as the residual value between observed and expected mass.
