# Supplementary material for: Foraging Behaviour of Juvenile Female New Zealand Sea Lions (Phocarctos hookeri) in Contrasting Environments
Source: PLoS One. 2013 May 6;8(5):e62728. doi: 10.1371/journal.pone.0062728 (PMC3646001; doi:10.1371/journal.pone.0062728)
Supplement: Table S1 — Morphometric, dive and satellite data available for individual juvenile female New Zealand sea lions ( Phocarctos hookeri ) at Auckland Islands (AI) and Otago Peninsula. BCI, body condition index. (DOC) [file pone.0062728.s002.doc]

Table S1. Morphometric, dive and satellite data available for individual juvenile female New Zealand sea lions (*Phocarctos hookeri*) at Auckland Islands (AI) and Otago Peninsula. BCI, body condition index. Mean ± standard error of mean (SEM).

| Study site | Animal id | Age | Mass (kg) | Length (cm) | BCI | Dive data | Satellite data |
| --- | --- | --- | --- | --- | --- | --- | --- |
| AI | 5876 | 2 | 55.5 | 152 | -14.6 |  | x |
| AI | 6111 | 2 | 56.0 | 138 | -0.7 |  | x |
| AI | 6463 | 2 | 73.5 | 146 | 9.1 |  | x |
| AI | 7458 | 2 | 57.0 | 140 | -1.6 |  | x |
| AI | 7610 | 2 | 54.0 | 140 | -4.6 | x | x |
| AI | 7445 | 2 | 53.0 | 138 | -3.7 | x | x |
| AI | 8023 | 2 | 54.0 | 135 | 0.1 | x | x |
| Mean ± SEM | |  | 57.6 ± 2.7 | 141.3 ± 2.2 | -2.3 ± 2.6 |  |  |
| AI | 5121 | 3 | 72.0 | 155 | -0.9 |  | x |
| AI | 5142 | 3 | 65.0 | 149 | -2.2 |  | x |
| AI | 6130 | 3 | 68.0 | 153 | -3.0 | x | x |
| AI | 5857 | 3 | 71.0 | 141 | 11.4 | x | x |
| AI | 5863 | 3 | 68.0 | 152 | -2.1 | x | x |
| AI | 5913 | 3 | 68.0 | 156 | -5.9 | x | x |
| AI | 6059 | 3 | 84.5 | 154 | 12.5 | x | x |
| AI | 6363 | 3 | 79.0 | 165 | -3.5 | x | x |
| AI | 6536 | 3 | 70.0 | 157 | -4.9 | x | x |
| AI | 7458 | 3 | 73.0 | 153 | -1.6 |  | x |
| AI | 7199 | 3 | 78.5 | 154 | 6.5 | x | x |
| AI | 7584 | 3 | 68.0 | 152 | -2.1 | x | x |
| Mean ± SEM | |  | 72.1 ± 1.7 | 153.4 ± 1.6 | 0.4 ± 1.8 |  |  |
| Otago | 3451 | 2 | 88.0 | 167 | 3.6 | x | x |
| Otago | 3455 | 2 | 79.0 | 154 | 7.0 | x | x |
| Otago | 3458 | 2 | 74.5 | 166 | -8.9 | x | x |
| Mean ± SEM | |  | 80.5 ± 4.0 | 162.3 ± 4.2 | 0.6 ± 4.8 |  |  |
| Otago | 2594 | 3 | 97.0 | 177 | 3.1 | x | x |
| Otago | 2591 | 3 | 95.5 | 175 | 3.5 | x | x |
| Mean ± SEM | |  | 96.3 ± 0.8 | 176.0 ± 1.0 | 3.3 ± 0.2 |  |  |
