# Supplementary material for: Foraging Behaviour of Juvenile Female New Zealand Sea Lions (Phocarctos hookeri) in Contrasting Environments
Source: PLoS One. 2013 May 6;8(5):e62728. doi: 10.1371/journal.pone.0062728 (PMC3646001; doi:10.1371/journal.pone.0062728)
Supplement: Table S2 — Results of linear mixed effects models run on juvenile New Zealand sea lion ( Phocarctos hookeri ) foraging trip characteristics: at sea duration, trip distance and maximum distance from study site. (DOC) [file pone.0062728.s003.doc]

Table S2. Results of linear mixed effects models run on juvenile New Zealand sea lion foraging trip characteristics: at sea duration, trip distance and maximum distance from study site.

| Trip characteristic | Model | Auckland Islands | Study site | Age | Mass | Study site:mass | Age:mass | k | AICc | ΔAICc | ωAICc |
| --- | --- | --- | --- | --- | --- | --- | --- | --- | --- | --- | --- |
| At sea duration (h; log transformed) | 2 | 3.540 | -1.073 |  |  |  |  | 5 | 359.552 | 0.000 | 0.392 |
|  | 6 | 3.604 | -1.193 |  | 0.075 |  |  | 6 | 361.279 | 1.728 | 0.165 |
|  | 5 | 3.548 | -1.085 | -0.027 |  |  |  | 6 | 361.482 | 1.931 | 0.149 |
|  | 8 | 3.833 | -1.589 | -0.181 | 0.274 |  |  | 7 | 362.056 | 2.504 | 0.112 |
|  | 9 | 3.623 | -1.160 |  | 0.096 | -0.091 |  | 7 | 363.207 | 3.655 | 0.063 |
|  | 12 | 3.860 | -1.586 | -0.214 | 0.274 |  | -0.068 | 8 | 363.677 | 4.125 | 0.050 |
|  | 11 | 3.842 | -1.562 | -0.179 | 0.286 | -0.061 |  | 8 | 364.021 | 4.470 | 0.042 |
|  | 13 | 3.850 | -1.644 | -0.233 | 0.247 | 0.135 | -0.098 | 9 | 365.582 | 6.031 | 0.019 |
|  | 7 | 3.063 |  | 0.222 | -0.377 |  |  | 6 | 369.435 | 9.883 | 0.003 |
|  | 4 | 3.166 |  |  | -0.275 |  |  | 5 | 370.242 | 10.690 | 0.002 |
|  | 10 | 3.090 |  | 0.189 | -0.375 |  | -0.065 | 7 | 371.192 | 11.640 | 0.001 |
|  | 1 | 3.306 |  |  |  |  |  | 4 | 372.313 | 12.761 | 0.001 |
|  | 3 | 3.290 |  | 0.069 |  |  |  | 5 | 374.058 | 14.506 | 0.000 |
| Trip distance (km; square root transformed) | 2 | 10.152 | -5.852 |  |  |  |  | 5 | 933.329 | 0.000 | 0.275 |
|  | 8 | 11.827 | -8.746 | -1.350 | 1.444 |  |  | 7 | 934.064 | 0.735 | 0.190 |
|  | 5 | 10.339 | -6.093 | -0.531 |  |  |  | 6 | 934.071 | 0.741 | 0.190 |
|  | 6 | 10.135 | -5.820 |  | -0.021 |  |  | 6 | 935.329 | 1.999 | 0.101 |
|  | 12 | 11.997 | -8.757 | -1.538 | 1.465 |  | -0.377 | 8 | 935.472 | 2.142 | 0.094 |
|  | 11 | 11.869 | -8.601 | -1.337 | 1.500 | -0.303 |  | 8 | 936.022 | 2.693 | 0.071 |
|  | 9 | 10.240 | -5.603 |  | 0.105 | -0.555 |  | 7 | 937.206 | 3.877 | 0.040 |
|  | 13 | 11.966 | -9.128 | -1.655 | 1.333 | 0.770 | -0.545 | 9 | 937.313 | 3.984 | 0.037 |
|  | 4 | 8.017 |  |  | -1.726 |  |  | 5 | 944.773 | 11.444 | 0.001 |
|  | 7 | 7.611 |  | 0.866 | -2.117 |  |  | 6 | 944.978 | 11.649 | 0.001 |
|  | 10 | 7.773 |  | 0.679 | -2.101 |  | -0.367 | 7 | 946.659 | 13.330 | 0.000 |
|  | 1 | 8.855 |  |  |  |  |  | 4 | 949.532 | 16.203 | 0.000 |
|  | 3 | 8.860 |  | -0.020 |  |  |  | 5 | 951.531 | 18.202 | 0.000 |
| Max distance from study site (km; power transformed) | 2 | 1.629 | -0.502 |  |  |  |  | 5 | -171.798 | 0.000 | 0.257 |
|  | 9 | 1.618 | -0.422 |  | -0.011 | -0.089 |  | 7 | -171.253 | 0.545 | 0.196 |
|  | 6 | 1.597 | -0.447 |  | -0.033 |  |  | 6 | -171.177 | 0.621 | 0.189 |
|  | 5 | 1.632 | -0.509 | -0.018 |  |  |  | 6 | -170.563 | 1.235 | 0.139 |
|  | 11 | 1.613 | -0.413 | 0.004 | -0.015 | -0.089 |  | 8 | -169.268 | 2.530 | 0.073 |
|  | 8 | 1.594 | -0.441 | 0.003 | -0.036 |  |  | 7 | -169.183 | 2.614 | 0.070 |
|  | 12 | 1.602 | -0.440 | -0.009 | -0.038 |  | -0.025 | 8 | -168.526 | 3.272 | 0.050 |
|  | 13 | 1.612 | -0.417 | 0.001 | -0.019 | -0.076 | -0.007 | 9 | -167.325 | 4.473 | 0.027 |
|  | 7 | 1.375 |  | 0.116 | -0.221 |  |  | 6 | -156.108 | 15.690 | 0.000 |
|  | 10 | 1.386 |  | 0.102 | -0.221 |  | -0.028 | 7 | -154.964 | 16.834 | 0.000 |
|  | 4 | 1.428 |  |  | -0.168 |  |  | 5 | -146.051 | 25.746 | 0.000 |
|  | 1 | 1.517 |  |  |  |  |  | 4 | -132.457 | 39.340 | 0.000 |
|  | 3 | 1.511 |  | 0.024 |  |  |  | 5 | -130.713 | 41.084 | 0.000 |
