# Supplementary material for: Foraging Behaviour of Juvenile Female New Zealand Sea Lions (Phocarctos hookeri) in Contrasting Environments
Source: PLoS One. 2013 May 6;8(5):e62728. doi: 10.1371/journal.pone.0062728 (PMC3646001; doi:10.1371/journal.pone.0062728)
Supplement: Table S3 — Results of linear mixed effects models run on juvenile New Zealand sea lion ( Phocarctos hookeri ) dive characteristics: mean and maximum dive depth and mean and maximum dive duration. (DOC) [file pone.0062728.s004.doc]

Table S3. Results of linear mixed effects models run on juvenile New Zealand sea lion dive characteristics: mean and maximum dive depth and mean and maximum dive duration.

| Trip characteristic | Model | Auckland Islands | Study site | Age | Mass | Study site:mass | Age:mass | k | AICc | ΔAICc | ωAICc |
| --- | --- | --- | --- | --- | --- | --- | --- | --- | --- | --- | --- |
| Dive depth (m; log transformed) | 2 | 4.237 | -1.540 |  |  |  |  | 5 | 138488.658 | 0.000 | 0.353 |
|  | 6 | 4.207 | -1.494 |  | -0.034 |  |  | 6 | 138490.184 | 1.526 | 0.165 |
|  | 9 | 4.175 | -1.511 |  | -0.071 | 0.138 |  | 7 | 138490.619 | 1.960 | 0.133 |
|  | 5 | 4.239 | -1.545 | -0.007 |  |  |  | 6 | 138490.628 | 1.970 | 0.132 |
|  | 8 | 4.127 | -1.366 | 0.057 | -0.100 |  |  | 7 | 138491.530 | 2.872 | 0.084 |
|  | 11 | 4.118 | -1.412 | 0.043 | -0.117 | 0.126 |  | 8 | 138492.226 | 3.568 | 0.059 |
|  | 12 | 4.109 | -1.353 | 0.083 | -0.102 |  | 0.038 | 8 | 138492.541 | 3.883 | 0.051 |
|  | 13 | 4.112 | -1.394 | 0.059 | -0.114 | 0.094 | 0.018 | 9 | 138494.091 | 5.433 | 0.023 |
|  | 7 | 3.347 |  | 0.496 | -0.729 |  |  | 6 | 138514.125 | 25.467 | 0.000 |
|  | 10 | 3.328 |  | 0.531 | -0.723 |  | 0.061 | 7 | 138515.524 | 26.866 | 0.000 |
|  | 4 | 3.531 |  |  | -0.534 |  |  | 5 | 138533.512 | 44.853 | 0.000 |
|  | 1 | 3.784 |  |  |  |  |  | 4 | 138542.170 | 53.511 | 0.000 |
|  | 3 | 3.739 |  | 0.238 |  |  |  | 5 | 138542.342 | 53.684 | 0.000 |
| Maximum dive depth (m; log transformed) | 2 | 5.021 | -1.580 |  |  |  |  | 5 | 490.544 | 0.000 | 0.279 |
|  | 6 | 5.135 | -1.718 |  | 0.084 |  |  | 6 | 490.783 | 0.239 | 0.248 |
|  | 5 | 5.011 | -1.549 | 0.056 |  |  |  | 6 | 491.251 | 0.707 | 0.196 |
|  | 9 | 5.102 | -1.692 |  | 0.060 | 0.047 |  | 7 | 492.645 | 2.101 | 0.098 |
|  | 8 | 5.150 | -1.739 | -0.009 | 0.094 |  |  | 7 | 492.776 | 2.232 | 0.092 |
|  | 11 | 5.115 | -1.709 | -0.007 | 0.068 | 0.047 |  | 8 | 494.640 | 4.096 | 0.036 |
|  | 12 | 5.144 | -1.727 | -0.008 | 0.087 |  | -0.010 | 8 | 494.731 | 4.187 | 0.034 |
|  | 13 | 5.017 | -1.587 | 0.003 | -0.021 | 0.140 | -0.049 | 9 | 496.155 | 5.611 | 0.017 |
|  | 7 | 3.943 |  | 0.552 | -0.671 |  |  | 6 | 511.878 | 21.334 | 0.000 |
|  | 10 | 3.957 |  | 0.529 | -0.677 |  | -0.044 | 7 | 513.542 | 22.998 | 0.000 |
|  | 4 | 4.200 |  |  | -0.463 |  |  | 5 | 531.870 | 41.326 | 0.000 |
|  | 3 | 4.450 |  | 0.297 |  |  |  | 5 | 537.796 | 47.252 | 0.000 |
|  | 1 | 4.522 |  |  |  |  |  | 4 | 538.283 | 47.739 | 0.000 |
| Dive duration (min) | 2 | 3.237 | -1.417 |  |  |  |  | 5 | 591517.519 | 0.000 | 0.269 |
|  | 6 | 3.343 | -1.581 |  | 0.122 |  |  | 6 | 591517.815 | 0.296 | 0.232 |
|  | 5 | 3.202 | -1.354 | 0.090 |  |  |  | 6 | 591517.854 | 0.335 | 0.228 |
|  | 8 | 3.280 | -1.479 | 0.045 | 0.070 |  |  | 7 | 591519.694 | 2.174 | 0.091 |
|  | 9 | 3.333 | -1.586 |  | 0.111 | 0.041 |  | 7 | 591519.775 | 2.256 | 0.087 |
|  | 12 | 3.302 | -1.496 | 0.015 | 0.073 |  | -0.043 | 8 | 591521.332 | 3.813 | 0.040 |
|  | 11 | 3.278 | -1.490 | 0.042 | 0.066 | 0.029 |  | 8 | 591521.674 | 4.155 | 0.034 |
|  | 13 | 3.310 | -1.578 | -0.032 | 0.053 | 0.180 | -0.082 | 9 | 591522.871 | 5.352 | 0.019 |
|  | 7 | 2.434 |  | 0.521 | -0.612 |  |  | 6 | 591530.229 | 12.710 | 0.000 |
|  | 10 | 2.440 |  | 0.511 | -0.613 |  | -0.017 | 7 | 591532.201 | 14.682 | 0.000 |
|  | 4 | 2.628 |  |  | -0.407 |  |  | 5 | 591544.580 | 27.061 | 0.000 |
|  | 3 | 2.764 |  | 0.304 |  |  |  | 5 | 591546.981 | 29.462 | 0.000 |
|  | 1 | 2.820 |  |  |  |  |  | 4 | 591548.257 | 30.738 | 0.000 |
| Maximum dive duration (min) | 6 | 6.214 | -2.002 |  | 0.650 |  |  | 6 | 2485.821 | 0.000 | 0.267 |
|  | 9 | 6.104 | -1.968 |  | 0.558 | 0.277 |  | 7 | 2487.508 | 1.687 | 0.115 |
|  | 13 | 6.054 | -2.034 | -0.371 | 0.337 | 1.119 | -0.437 | 9 | 2487.573 | 1.752 | 0.111 |
|  | 5 | 5.264 | -0.686 | 0.443 |  |  |  | 6 | 2487.677 | 1.856 | 0.105 |
|  | 8 | 6.323 | -2.153 | -0.063 | 0.720 |  |  | 7 | 2487.797 | 1.975 | 0.099 |
|  | 3 | 5.007 |  | 0.543 |  |  |  | 5 | 2488.294 | 2.473 | 0.077 |
|  | 12 | 6.365 | -2.122 | -0.163 | 0.698 |  | -0.169 | 8 | 2488.837 | 3.015 | 0.059 |
|  | 7 | 4.859 |  | 0.627 | -0.209 |  |  | 6 | 2489.200 | 3.379 | 0.049 |
|  | 11 | 6.244 | -2.167 | -0.083 | 0.649 | 0.285 |  | 8 | 2489.465 | 3.644 | 0.043 |
|  | 2 | 5.447 | -0.960 |  |  |  |  | 5 | 2490.074 | 4.253 | 0.032 |
|  | 10 | 4.925 |  | 0.507 | -0.218 |  | -0.178 | 7 | 2490.357 | 4.536 | 0.028 |
|  | 1 | 5.129 |  |  |  |  |  | 4 | 2492.241 | 6.419 | 0.011 |
|  | 4 | 5.136 |  |  | 0.011 |  |  | 5 | 2494.238 | 8.417 | 0.004 |
